# Reduced micromorphic model in orthogonal curvilinear coordinates and its application to a metamaterial hemisphere

**DOI:** 10.1038/s41598-020-59696-8

**Published:** 2020-02-18

**Authors:** A. R. El Dhaba

**Affiliations:** Department of Mathematics, Faculty of Science, Damanhour University, Damanhour, Egypt

**Keywords:** Nanoscale materials, Structural materials, Mechanical engineering, Nanoscale materials

## Abstract

The reduced micromorphic model (RMM) is used to study the effect of the applied force on a hemisphere made of phononic crystals that belongs to the metamaterials group. The strain tensor, the micro-strain tensor and the coupling between them are the kinematic relations used to measure the deformation and micro-deformation of the representative volume element of these materials. The free energy function, the constitutive relations, the field equations, and the boundary conditions are presented firstly in the Cartesian coordinate. Then, the orthogonal curvilinear coordinates are introduced as a general coordinate to describe the physical quantities included in the RMM. The spherical coordinates are deduced as a special case from the curvilinear coordinates to study the deformation and micro-deformation for the hemisphere. The kinematic relations and the governing equations of the model are considered to changing with the radius of the hemisphere only. The analytical solutions of the field equations are also obtained by using the Frobenius series satisfying the given boundary conditions and consequently the value of the physical constants of the problem is determined. Numerical applications for the obtained results are introduced with discussion. The results showed that the displacement has a greater effect rather than the micro-strain, when it is measured relative to the classical physical quantities while the micro-strain has a greater effect rather than the displacement, when it is measured relative to the nanoscale physical quantities.

## Introduction

Metamaterials are fabricated engineering materials with periodic internal structures. These materials have special properties that do not exist in other natural materials. They can be designed to prohibit the propagation of elastic waves in the bandgap frequency range effectively. This property has many potential applications in the vibration and noise reduction areas. Besides this property, there are many applications for metamaterials in the fields of technology, industrial engineering, telecommunication equipment, optical filters, medical devices and mobile communication systems and others.

The main idea of constructing metamaterials is attributed to Sir J.C. Bose in 1898. He suggested the idea of the existence of artificial materials by carrying the microwave experiment on twisted structures^1,2^. Winston E. Kock^3,4^, used the optical radio waves properties to develop new type of antennas made of metamaterial. V. G. Veselago^5^, introduced a theoretical model that predicts the propagation of electromagnetic waves in left-handed materials. During the last few decades, more attention was given to metamaterials. This is because of their important applications in recent industries. Theoretical formulation and mechanical applications can be found in refs. ^6–9^.

The idea of building metamaterials was extended to include elastic and acoustic waves by Ding *et al*.^10^, where the authors proposed metamaterials with simultaneously negative bulk modulus and mass density. Wu *et al*.^11^ proposed a new type of elastic metamaterials made from fluid-solid components possessing negative shear modulus and negative mass density for a large frequency domain. To study the new phenomena at the micro-scale, many theories have been introduced to modify the classical theory of continuum mechanics by adding an additional degree of freedom. This describes the micro-effects relative to the macro-effects. Micropolar theory (Cosserat theory)^12^, couple stress theory^13^, micromorphic theory^14^, microstructure theory^15^, and micropolar theory^16^ are extensions of the classical field theory to microscopic field theory in space and time scales. Among these theories, the micromorphic theory developed by Eringen and Suhubi^17,18^ and Eringen^19,20^ is an extension of the classical field theories to microscopic time and space scales. In this new theory, the concept of material point used in classical field theory is replaced by a representative volume element (RVE) that can move, deform, rotate and stretch. Also, the molecules that constitute the RVE can deform and rotate. Moreover, the Micromorphic model can be reduced to yield other models. This can be achieved by imposing internal constraints prescribed on the micro-deformation^21^. When the micro-deformation is constrained to be pure rotation, micropolar theory is obtained. Also, when the micro-deformation coincides with the deformation gradient, the second gradient theory is obtained. Classical field theory is retrieved when the displacement field is a linear transformation and the micro-deformation vanishes.

Among the important phenomena that characterize the micromorphic model: the size-dependent effect, 18 length scale parameters for the isotropic case, two types of wave propagation, acoustic and optic waves, and bandgaps in the frequency domain.

Micromorphic theory provides basic field equations and constitutive relations for large classes of materials such as artificial materials, porous materials, composites, polymers, crystals, etc., whose REV possesses independent degrees of freedom and described boundary conditions for each boundary-value problem. Neff *et al*.^22^, proposed a relaxed linear elastic micromorphic model to reduce the number of material parameters. The number of the materials parameters is reduced to 9, the coupling between materials parameters is reduced to 4 dependent +3 independent. Also, the types of waves are acoustic waves, optic waves, and standing waves for the relaxed micromorphic model. Shaat^23^ reduced the number of the material parameters to 8, the coupling between materials parameters are 8 dependent + 0 independent. Three types of waves are obtained. Shaat and El Dhaba, in^24^ used the so-called reduced micromorphic model (RMM) to study the equivalent shear modulus for composite metamaterials.

The main objective of this article is to use the generalized curvilinear coordinates to introduce a novel description for the reduced micromorphic model with extension to spherical coordinates and applications in engineering and mechanics. The importance of this study arises from its potential applications in spongy materials, granular materials, as well as for materials used in lubrication and fluidization for the mechanical parts in cars, trucks and internal structure of engines. The paper is organized as follows: Section 2 is devoted to introducing the RMM in Cartesian coordinates with complete description of the kinematic relations, the definition of the strain energy function as well as the description of the constitutive relations, the field equations and the boundary conditions. Section 3 describes the mathematical formulas of the model in general curvilinear coordinates. An application for the model in spherical polar coordinates is presented in Section 4. Finally, in Section 5 we find the solution of the considered problem. The obtained results are presented in Section 6 with detailed discussion. Finally, the main findings of this study are presented in Section 7.

## The RMM in Cartesian Coordinates

Because the classical theories of continuum mechanics do not have the ability to represent the nanoscale phenomena, the reduced micromorphic model (RMM)^23^ is introduced to study such phenomena at the micro-scale level. The RMM introduces the micro-strain tensor as an unknown measure, besides the displacement components. Also, it introduces the coupling between the strain tensor and the micro-strain tensor as a coupling measure with elimination of the repeated effects. In addition to its ability to reduce the material parameters, the RMM generates more field equations and reduces the order of the partial differential equations of the model. These properties make it possible to obtain analytical solutions for the physical state variables of the model. Materials whose behavior is described by this model are called “multiscale materials” or micromorphic materials.

In RMM, the kinematical variables are defined as follows:

1 $${\varepsilon }_{ij}=\frac{1}{2}({u}_{i,j}+{u}_{j,i}),\,{\gamma }_{ij}={\varepsilon }_{ij}-{s}_{ij},\,{\chi }_{ijk}={s}_{jk,i},\,{\chi }_{ijj=0.}$$

where ${\varepsilon }_{ij}(\,=\,{\varepsilon }_{ji})$ denote the classical strain tensor, ${s}_{ij}(\,=\,{s}_{ji})$ is the micro-strain tensor, ${\gamma }_{ij}(\,=\,{\gamma }_{ji})$ is the coupling between the micro-strain ${s}_{ij}$ and the macro-strain ${\varepsilon }_{ij}$ and ${\chi }_{ijk}(\,=\,{\chi }_{ikj})$ is the gradient of the micro-strain tensor ${s}_{ij}$.

The equations of motion can be derived by using variational methods and are given as:

2 $$\begin{array}{c}{\tau }_{ji,j}+{f}_{i}=\rho {\ddot{u}}_{i},\\ {m}_{ijk,i}+{\tau }_{jk}-{t}_{jk}+{H}_{jk}={\rho }_{m}J{\ddot{s}}_{jk},\end{array}\}$$

the natural boundary conditions are

$${n}_{j}{\tau }_{ji}={\bar{t}}_{i},\,{n}_{i}{m}_{ijk}={\bar{m}}_{jk},$$

where $\rho$ is the mass density of the macro-scale material, ${\rho }_{m}$ is the mass density of the material particle, and *J* denotes a microinertia density per unit mass, ${f}_{i}$ and ${H}_{jk}$ are the body force and the body higher-order-moments respectively.

The constitutive relations are related to the free energy function by:

3 $${t}_{ij}=\frac{\partial W}{\partial {s}_{ij}},\,{\tau }_{ij}=\frac{\partial W}{\partial {\gamma }_{ij}},\,{m}_{ijk}=\frac{\partial W}{\partial {\chi }_{ijk}},$$

where $W=W({\varepsilon }_{ij},{\gamma }_{ij},{\chi }_{ijk})$ is the free energy function in terms of internal variables, ${t}_{ij}$ is the micro-stress tensor, ${\tau }_{ij}$ can be defined as the residual stress and ${m}_{ijk}$ is a higher order micro-stress tensor.

According to^23,24^, the free energy is taken in the form

4 $$\begin{array}{rcl}W & = & \frac{1}{2}{\lambda }_{m}{s}_{ii}{s}_{jj}+{\mu }_{m}{s}_{ij}{s}_{ij}+\frac{1}{2}\lambda {\gamma }_{ii}{\gamma }_{jj}+\mu {\gamma }_{ij}{\gamma }_{ij}+{\lambda }_{c}{\gamma }_{ii}{s}_{jj}+2{\mu }_{c}{\gamma }_{ij}{s}_{ij}\\  &  & +\,\frac{1}{2}{\lambda }_{m}{\ell }_{1}^{2}({\chi }_{iik}{\chi }_{jjk}+{\chi }_{ijk}{\chi }_{jik})+\frac{1}{2}{\mu }_{m}{\ell }_{2}^{2}{\chi }_{ijk}{\chi }_{ijk},\end{array}$$

where ${\lambda }_{m}$ and *μ*_*m*_ are the elastic moduli of the microstructure, *λ* and *μ* are the elastic moduli of the confined material between two particles, *λ*_*c*_ and *μ*_*c*_ are two elastic moduli accounting for the coupling between the micro-strain and the macro-strain, ${\ell }_{1}$ and ${\ell }_{2}$ are length scale parameters. Such a medium is composed of deformed molecules and have twelve degree of freedom: three translational, three rotational and six micro-deformations^25^ and^26^. Many authors introduce a simplified version of the mathematical model for such materials in order to reduce the material parameters^22^ and^23^.

Substitute Eq. (4) into Eq. (3) to get the constitutive relations as:

5 $$\begin{array}{rcl}{t}_{ij} & = & {\lambda }_{m}{\delta }_{ij}{s}_{qq}+2{\mu }_{m}{s}_{ij}+{\lambda }_{c}{\delta }_{ij}{\gamma }_{qq}+2{\mu }_{c}{\gamma }_{ij},\\ {\tau }_{ij} & = & \lambda {\delta }_{ij}{\gamma }_{qq}+2\mu {\gamma }_{ij}+{\lambda }_{c}{\delta }_{ij}{s}_{qq}+2{\mu }_{c}{s}_{ij},\\ {m}_{ijk} & = & {\lambda }_{m}{\ell }_{1}^{2}{\delta }_{ij}{\chi }_{qqk}+{\mu }_{m}{\ell }_{2}^{2}{\chi }_{ijk}+{\lambda }_{m}{\ell }_{1}^{2}{\chi }_{jik}.\end{array}\}$$

## The RMM Model in Orthogonal Curvilinear Coordinates

In this section, we introduce a theoretical method to obtain the governing equations, boundary conditions and the constitutive relations for the RMM in orthogonal curvilinear coordinates. The main idea of the method depends on two concepts mentioned by Eringen^27^. We introduce the main rules for the derivatives of the covariant and contravariant vectors, and the second and higher orders contravariant and mixed tensors as follows:

6 $$\begin{array}{rcl}{A}_{;j}^{i} & = & {A}_{,j}^{i}+{{\Gamma }}_{qj}^{i}{A}^{q},\\ {B}_{;i}^{jk} & = & {B}_{,i}^{jk}+{{\Gamma }}_{qi}^{j}{B}^{kq}+{{\Gamma }}_{qi}^{k}{B}^{qj}\\ {C}_{k;i}^{j} & = & {C}_{k,i}^{j}+{{\Gamma }}_{qi}^{j}{C}_{k}^{q}-{{\Gamma }}_{ik}^{q}{C}_{q}^{j}\\ {D}_{;p}^{ijk} & = & {D}_{,p}^{ijk}+{{\Gamma }}_{pq}^{i}{D}^{qjk}+{{\Gamma }}_{pq}^{j}{D}^{iqk}+{{\Gamma }}_{pq}^{k}{D}^{ijq},\\ {E}_{k;p}^{ji} & = & {E}_{k,p}^{ji}+{{\Gamma }}_{pq}^{j}{E}_{k}^{qi}+{{\Gamma }}_{pq}^{i}{E}_{k}^{jq}-{{\Gamma }}_{kp}^{q}{E}_{q}^{ji},\\ {F}_{jk;l}^{i} & = & {F}_{jk,l}^{i}+{{\Gamma }}_{lm}^{i}{F}_{jk}^{m}-{{\Gamma }}_{lj}^{m}{F}_{mk}^{i}-{{\Gamma }}_{lk}^{m}{F}_{jm}^{i},\end{array}\}$$

where *A*^*i*^ are the vector contravariant components, *B*^*jk*^- the second rank tensor contravariant components, ${C}_{k}^{j}$ - the components of second rank mixed tensor, *D*^*ijk*^- the contravariant components of third rank tensor and ${E}_{k}^{ji}$, ${F}_{jk}^{i}$ are the components of the third rank mixed tensors. ${{\Gamma }}_{jk}^{i}$ are the known Christoffel symbols of the second kind satisfying ${{\Gamma }}_{jk}^{i}={{\Gamma }}_{kj}^{i}$ and defined in orthogonal curvilinear coordinates as follows^28^:

7 $${{\Gamma }}_{jk}^{i}=\frac{1}{2}{g}^{ia}({g}_{aj,k}+{g}_{ak,j}-{g}_{jk,a})$$

where *g*_*ij*_ are the metric tensor components.

Following the two concepts proposed by Eringen^27^, we replace the partial differentiation (,) with the covariant differentiation (;) taking in consideration the repeated indices summation rule.

Therefore, the kinematic relations (1) can be written in mixed tensor form as follows:

8 $${\varepsilon }_{j}^{i}=\frac{1}{2}({u}_{;j}^{i}+{g}_{jm}{g}^{in}{u}_{;n}^{m}),\,{\gamma }_{j}^{i}={\varepsilon }_{j}^{i}-{s}_{j}^{i},\,{\chi }_{k}^{ij}={s}_{k;i}^{j},$$

Following^29,30^, we can use the following relation in the second term:

$${u}_{;i}^{j}={g}_{jm}{g}^{in}{u}_{;n}^{m}.$$

Using Eq. (6), Eq. (8) can be written as:

9 $$\begin{array}{rcl}{\varepsilon }_{j}^{i} & = & \frac{1}{2}({u}_{,j}^{i}+{\varGamma }_{qj}^{i}{u}^{q}+{g}_{jm}{g}^{in}({u}_{,n}^{m}+{\varGamma }_{qn}^{m}{u}^{q})),\\ {\gamma }_{j}^{i} & = & \frac{1}{2}({u}_{,j}^{i}+{\varGamma }_{qj}^{i}{u}^{q}+{g}_{jm}{g}^{in}({u}_{,n}^{m}+{\varGamma }_{qn}^{m}{u}^{q}))-{s}_{j}^{i},\\ {\chi }_{k}^{ij} & = & {s}_{k;i}^{j}={s}_{k,i}^{j}+{\varGamma }_{ik}^{q}{s}_{q}^{j}-{\varGamma }_{qi}^{j}{s}_{k}^{q}.\end{array}\}$$

The constitutive relations in terms of the contravariant components are given by:

10 $$\begin{array}{rcl}{t}_{j}^{i} & = & {\lambda }_{m}{\delta }_{j}^{i}{s}_{q}^{q}+2{\mu }_{m}{s}_{j}^{i}+{\lambda }_{c}{\delta }_{j}^{i}{\gamma }_{q}^{q}+2{\mu }_{c}{\gamma }_{j}^{i},\\ {\tau }_{j}^{i} & = & \lambda {\delta }_{j}^{i}{\gamma }_{q}^{q}+2\mu {\gamma }_{j}^{i}+{\lambda }_{c}{\delta }_{j}^{i}{s}_{q}^{q}+2{\mu }_{c}{s}_{j}^{i},\\ {m}_{k}^{ij} & = & {\lambda }_{m}{\ell }_{1}^{2}{\delta }_{j}^{i}{\chi }_{k}^{qq}+{\mu }_{m}{\ell }_{2}^{2}{\chi }_{k}^{ij}+{\lambda }_{m}{\ell }_{1}^{2}{\chi }_{k}^{ji}.\end{array}\}$$

Following^27–30^, the equations of motion in the RMM model may be written in the form:

11 $$\begin{array}{rcl}{\tau }_{i;j}^{j}+{f}^{j} & = & \rho {\ddot{u}}^{j},\\ {m}_{k;i}^{ij}+{\tau }_{k}^{j}-{t}_{k}^{j}+{H}_{k}^{j} & = & {\rho }_{m}J{\ddot{s}}_{k}^{j},\end{array}\}$$

where (;) denotes covariant differentiation.

Using Eq. (6), the covariant derivatives of the second and third rank tensors are:

12 $$\begin{array}{rcl}{\tau }_{i;j}^{j} & = & {\tau }_{i,j}^{j}+{{\Gamma }}_{qj}^{j}{\tau }_{i}^{q}-{{\Gamma }}_{ji}^{q}{\tau }_{q}^{j},\\ {m}_{k;i}^{ij} & = & {m}_{k,i}^{ij}+{{\Gamma }}_{iq}^{i}{m}_{k}^{qj}+{{\Gamma }}_{iq}^{j}{m}_{k}^{iq}-{{\Gamma }}_{ki}^{q}{m}_{q}^{ij}.\end{array}\}$$

Neglecting external forces and moments, substituting Eq. (12) into Eq. (11) one gets:

13 $$\begin{array}{rcl}{\tau }_{i,j}^{j}+{{\Gamma }}_{qj}^{j}{\tau }_{i}^{q}-{{\Gamma }}_{ji}^{q}{\tau }_{q}^{j}+{f}^{j} & = & \rho {\ddot{u}}^{j},\\ {m}_{k,i}^{ij}+{{\Gamma }}_{iq}^{i}{m}_{k}^{qj}+{{\Gamma }}_{iq}^{j}{m}_{k}^{iq}-{{\Gamma }}_{ki}^{q}{m}_{q}^{ij}+{\tau }_{k}^{j}-{t}_{k}^{j}+{H}_{k}^{j} & = & {\rho }_{m}J{\ddot{s}}_{k}^{j}.\end{array}\}$$

According to^30^ and^31^, the vector and tensor physical components are

14 $$\begin{array}{rcl}{u}_{(i)} & = & \sqrt{{g}_{\underline{i}\underline{i}}}{u}^{i},\,{\varepsilon }_{(i)(j)}=\sqrt{\frac{{g}_{\underline{i}\underline{i}}}{{g}_{\underline{j}\underline{j}}}}{\varepsilon }_{j}^{i},\\ {\tau }_{(i)(j)} & = & \sqrt{\frac{{g}_{\underline{i}\underline{i}}}{{g}_{\underline{j}\underline{j}}}}{\tau }_{j}^{i},\,{m}_{(i)(j)(k)}=\sqrt{\frac{{g}_{\underline{i}\underline{i}}{g}_{\underline{j}\underline{j}}}{{g}_{\underline{k}\underline{k}}}}{m}_{k}^{ij},\end{array}\}$$

where $\sqrt{{g}_{\underline{i}\underline{i}}}$ are the Lamé coefficients. Also, the relation between the contravariant and covariant components are:

$${u}^{(i)}={g}^{im}{u}_{(m)},\,{u}_{(i)}={g}_{im}{u}^{(m)}.$$

To obtain the kinematic relations, the constitutive relations and the field equations in terms of the physical quantities we substitute Eq. (14) into Eqs. (9), (10) and (13):

15 $$\begin{array}{rcl}{\varepsilon }_{(i)(j)} & = & \frac{1}{2}\sqrt{\frac{{g}_{\underline{i}\underline{i}}}{{g}_{\underline{j}\underline{j}}}}({(\frac{{u}_{(i)}}{\sqrt{{g}_{\underline{i}\underline{i}}}})}_{,j}+{{\Gamma }}_{qj}^{i}\frac{{u}^{q}}{\sqrt{{g}_{qq}}}+{g}_{jm}{g}^{in}({(\frac{{u}_{(m)}}{\sqrt{{g}_{\underline{m}\underline{m}}}})}_{,n}+{{\Gamma }}_{qn}^{m}\frac{{u}_{(q)}}{\sqrt{{g}_{\underline{q}\underline{q}}}})),\\ {\gamma }_{(i)(j)} & = & \frac{1}{2}\sqrt{\frac{{g}_{\underline{i}\underline{i}}}{{g}_{\underline{j}\underline{j}}}}({(\frac{{u}_{(i)}}{\sqrt{{g}_{\underline{i}\underline{i}}}})}_{,j}+{g}_{jm}{g}^{in}{(\frac{{u}_{(m)}}{\sqrt{{g}_{\underline{m}\underline{m}}}})}_{,n})-\sqrt{\frac{{g}_{\underline{j}\underline{j}}}{{g}_{\underline{i}\underline{i}}}}{s}_{(i)(j)},\\ {\chi }_{(i)(j)(k)} & = & {(\sqrt{\frac{{g}_{\underline{k}\underline{k}}}{{g}_{\underline{j}\underline{j}}}}{s}_{(j)(k)})}_{,i}+\sqrt{\frac{{g}_{\underline{q}\underline{q}}}{{g}_{\underline{j}\underline{j}}}}{{\Gamma }}_{ik}^{q}{s}_{(j)(q)}-\sqrt{\frac{{g}_{\underline{k}\underline{k}}}{{g}_{\underline{q}\underline{q}}}}{{\Gamma }}_{qi}^{j}{s}_{(k)(q)},\end{array})$$

16 $$\begin{array}{rcl}{t}_{(i)(j)} & = & {\lambda }_{m}{\delta }_{(i)(j)}{s}_{(q)(q)}+2{\mu }_{m}{s}_{(i)(j)}+{\lambda }_{c}{\delta }_{(i)(j)}{\gamma }_{(q)(q)}+2{\mu }_{c}{\gamma }_{(i)(j)},\\ {\tau }_{(i)(j)} & = & \lambda {\delta }_{(i)(j)}{\gamma }_{(q)(q)}+2\mu {\gamma }_{(i)(j)}+{\lambda }_{c}{\delta }_{(i)(j)}{s}_{(q)(q)}+2{\mu }_{c}{s}_{(i)(j)},\\ {m}_{(i)(j)(k)} & = & {\lambda }_{m}{\ell }_{1}^{2}{\delta }_{(i)(j)}\sqrt{\frac{{g}_{\underline{j}\underline{j}}{g}_{\underline{j}\underline{j}}}{{g}_{\underline{q}\underline{q}}{g}_{\underline{q}\underline{q}}}}{\chi }_{(q)(q)(k)}+{\lambda }_{m}{\ell }_{1}^{2}{\chi }_{(j)(i)(k)}+{\mu }_{m}{\ell }_{2}^{2}{\chi }_{(i)(j)(k)}.\end{array}\}$$

and

17 $$\begin{array}{c}{(\sqrt{\frac{{g}_{\underline{ii}}}{{g}_{\underline{jj}}}}{\tau }_{(i)(j)})}_{,j}+{{\Gamma }}_{qj}^{j}\sqrt{\frac{{g}_{\underline{ii}}}{{g}_{\underline{qq}}}}{\tau }_{(q)(i)}-{{\Gamma }}_{ji}^{q}\sqrt{\frac{{g}_{\underline{qq}}}{{g}_{\underline{jj}}}}{\tau }_{(j)(q)}+\frac{{f}_{(j)}}{\sqrt{{g}_{\underline{jj}}}}=\frac{\rho }{\sqrt{{g}_{\underline{jj}}}}{\ddot{u}}_{(j)},\\ {(\sqrt{\frac{{g}_{\underline{kk}}}{{g}_{\underline{ii}}{g}_{\underline{jj}}}}{m}_{(i)(j)(k)})}_{,i}+{{\Gamma }}_{iq}^{i}\sqrt{\frac{{g}_{\underline{kk}}}{{g}_{\underline{qq}}{g}_{\underline{jj}}}}{m}_{(q)(j)(k)}+{{\Gamma }}_{iq}^{j}\sqrt{\frac{{g}_{\underline{kk}}}{{g}_{\underline{ii}}{g}_{\underline{qq}}}}{m}_{(i)(q)(k)},\\ -{\varGamma }_{ki}^{q}\sqrt{\frac{{g}_{\underline{qq}}}{{g}_{\underline{ii}}{g}_{\underline{jj}}}}{m}_{(i)(j)(q)}+\sqrt{\frac{{g}_{\underline{kk}}}{{g}_{\underline{jj}}}}{\tau }_{(j)(k)}-\sqrt{\frac{{g}_{\underline{kk}}}{{g}_{\underline{jj}}}}{t}_{(j)(k)}\\ +\sqrt{\frac{{g}_{\underline{kk}}}{{g}_{\underline{jj}}}}{H}_{(j)(k)}=\rho mJ\sqrt{\frac{{g}_{\underline{kk}}}{{g}_{\underline{jj}}}}{\ddot{s}}_{(j)(k)},\end{array}\}$$

It should be noted that some authors write the physical components as mixed tensors, as in^29,30^, while others write the physical components as covariant tensors as in^32^.

## Half-space Involving Spherical Symmetry

**Figure 1:**
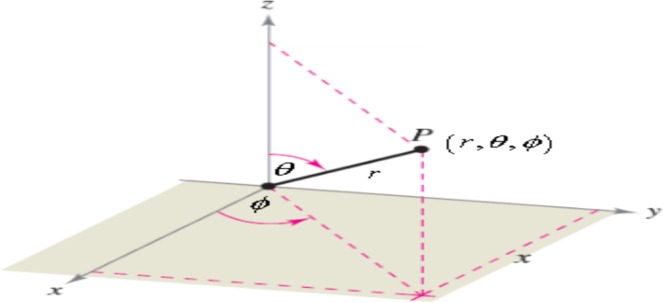
Spherical coordinates system.

Assume the an isotropic material occupying the half-space $x\ge 0$, in the *xyz*-plane containing a metamaterial hemisphere with radius *R*. Using the spherical coordinates (*r*, *θ*, $\varnothing$) (Fig. 1),

where,

18 $$x=r\,{\sin }(\theta ){\cos }(\varphi ),\,y=r\,{\sin }(\theta ){\sin }(\varphi ),\,z=r\,{\cos }(\theta ),$$

the metric tensor in spherical coordinates is defined as:

$${{\boldsymbol{g}}}_{{\boldsymbol{ij}}}=[\begin{array}{ccc}1 & 0 & 0\\ 0 & {r}^{2} & 0\\ 0 & 0 & {r}^{2}{si}{{n}}^{2}\theta \end{array}],$$

and the components of the metric tensor are:

19 $${g}_{11}=1,\,{g}_{22}={r}^{2}\,{g}_{33}={r}^{2}{si}{{n}}^{2}\theta ,$$

the Christoffel symbol of the second kind are:

20 $$\begin{array}{rcl}{\varGamma }_{22}^{1} & = & -r,\,{{\Gamma }}_{33}^{1}=-\,r{si}{{n}}^{2}\theta ,\,{{\Gamma }}_{33}^{2}=-\,{\sin }\,\theta \,{\cos }\,\theta ,\\ {\varGamma }_{12}^{2} & = & {{\Gamma }}_{31}^{3}=\frac{1}{r},\,{{\Gamma }}_{32}^{3}=\,{\cot }\,\theta ,\end{array}\}$$

without any body forces and body higher-order-moments and under the assumption of central symmetry, it is expected that the components of the displacement and micro-strain fields along $\theta$ and $\phi$ vanish, hence

21 $${\rm{u}}=({u}_{r}(r),0,0),\,s=({s}_{rr}(r),0,0).$$

Substitute the Eqs. (19–21) into Eq. (15), to get the kinematic relations in spherical coordinates as follows

22 $$\begin{array}{rcl}{\varepsilon }_{rr} & = & {u}_{r,r},\,{\varepsilon }_{\theta \theta }={\varepsilon }_{\varphi \varphi }=\frac{{u}_{r}}{r},\\ {\gamma }_{rr} & = & {u}_{r,r}-{s}_{rr},\,{\gamma }_{\theta \theta }={\gamma }_{\varphi \varphi }=\frac{{u}_{r}}{r},\\ {\chi }_{rrr} & = & {s}_{rr,r}=0,\,{\chi }_{\theta \theta r}={\chi }_{\varphi \varphi r}=-\,\frac{{s}_{rr}}{r}.\end{array}\}$$

Similarly, substitute Eqs. (19–21) into Eq. (16) to get components of the micro-stress tensor in spherical coordinates:

23 $$\begin{array}{rcl}{t}_{rr} & = & ({a}_{1}-{a}_{2}){s}_{rr}+{a}_{2}{u}_{r,r}+2{\lambda }_{c}\frac{{u}_{r}}{r},\\ {t}_{\theta \theta } & = & {b}_{1}{s}_{rr}+{\lambda }_{c}{u}_{r,r}+2{\lambda }_{c}\frac{{u}_{r}}{r},\\ {t}_{\varphi \varphi } & = & {b}_{1}{s}_{rr}+{\lambda }_{c}{u}_{r,r}+2{\lambda }_{c}\frac{{u}_{r}}{r},\\ {t}_{r\theta } & = & {t}_{r\varphi }={t}_{\theta \varphi }=0,\end{array}\}$$

the components of the residual stress tensor in spherical coordinates:

24 $$\begin{array}{rcl}{\tau }_{rr} & = & -({a}_{3}-{a}_{2}){s}_{rr}+{a}_{3}{u}_{r,r}+2\lambda \frac{{u}_{r}}{r},\\ {\tau }_{\theta \theta } & = & -{b}_{2}{s}_{rr}+\lambda {u}_{r,r}+{b}_{3}\frac{{u}_{r}}{r},\\ {\tau }_{\varphi \varphi } & = & -{b}_{2}{s}_{rr}+\lambda {u}_{r,r}+{b}_{3}\frac{{u}_{r}}{r},\\ {\tau }_{r\theta } & = & {\tau }_{\theta \varphi }={\tau }_{\theta \varphi }=0,\end{array}\}$$

and the components of the higher order micro-stress tensor with ${\ell }_{1}=0$, in spherical coordinates:

25 $$\begin{array}{rcl}{m}_{\theta \theta r} & = & -{c}_{2}\frac{{s}_{rr}}{r},\,{m}_{\varphi \varphi r}=-\,{c}_{2}\frac{{s}_{rr}}{r},\\ {m}_{\theta r\theta } & = & {m}_{\varphi r\varphi }={m}_{\theta rr}={m}_{\varphi rr}=0,\end{array}\}$$

with

$$\begin{array}{rcl}{a}_{1} & = & {\lambda }_{m}+2{\mu }_{m},\,{a}_{2}={\lambda }_{c}+2{\mu }_{c},\,{a}_{3}=\lambda +2\mu \\ {b}_{1} & = & {\lambda }_{m}-{\lambda }_{c},\,{b}_{2}=\lambda -{\lambda }_{c},\,{b}_{3}=2(\lambda +\mu ),\\ {c}_{1} & = & 0,\,{c}_{2}={\mu }_{m}{\ell }_{2}^{2},\,{c}_{3}=\mu -{\mu }_{c}.\end{array}\}$$

Substituting Eqs. (19–20) into Eq. (13) and neglecting the dynamic effects, we get the field equations in static equilibrium as follows:

26 $$\begin{array}{c}r{\tau }_{rr,r}+2{\tau }_{rr}-{\tau }_{\theta \theta }-{\tau }_{\varphi \varphi }=0,\\ {m}_{\theta \theta r}+{m}_{\varphi \varphi r}-r{\tau }_{rr}+r{t}_{rr}=0.\end{array}\}$$

Substitute Eqs. (23–25) into Eq. (26) to obtain the field equations of a hemisphere made of elastic phononic material in spherical coordinate as follows:

27 $$\begin{array}{c}({a}_{2}-{a}_{3}){r}^{2}{s}_{rr,r}-4{c}_{3}r{s}_{rr}+{a}_{3}({r}^{2}{u}_{r,rr}+2r{u}_{r,r}-2{u}_{r})=0,\\ ({a}_{3}-2{a}_{2}+{a}_{1}){r}^{2}{s}_{rr}-2{c}_{2}{s}_{rr}-({a}_{3}-{a}_{2}){r}^{2}{u}_{r,r}-2{b}_{2}r{u}_{r}=0,\end{array}\}$$

subjected to the boundary conditions

28 $${{u}_{r}(r)|}_{r=0}=0,\,{{\tau }_{rr}(r)|}_{r=R}={q}_{r}.$$

**Figure 2:**
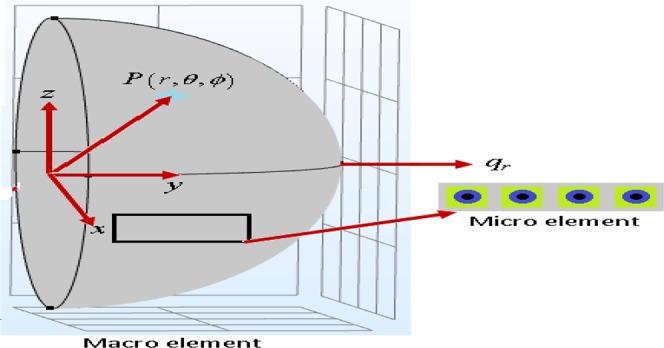
Hemisphere embedded in an isotropic half-space.

Figure 2. represents a hemisphere with radius *R* made of an elastic phononic material, the Cartesian coordinates are chosen at the lower surface of the hemisphere, we take the hemisphere fixed at origin of the coordinates and subjected to an external force in *r*-direction.

The hemisphere is described by

29 $$r=[0,R],\,\theta =[0,\,\pi ],\,\varphi =[0,\,\pi ].$$

## The Analytical Solution

Frobenius series is employed to get the analytical solution for the system of ordinary differential equations in (27) with the boundary conditions in Eq. (28). Since the point (0, 0, 0) is a regular point, one can express the functions *u*_*r*_ and *s*_*rr*_ in terms of the variable *r* as follows:

30 $$\begin{array}{cc}{u}_{r}(r)=\mathop{\sum }\limits_{n=0}^{\infty }\,{A}_{n}{r}^{n}, & {s}_{rr}(r)=\mathop{\sum }\limits_{n=0}^{\infty }\,{B}_{n}{r}^{n},\end{array}$$

where *A*_*n*_ and *B*_*n*_ are constants to be determined from the given boundary conditions.

Substituting the expressions for the two functions *u*_*r*_ and *s*_*rr*_ and their derivatives into Eq. (27), we obtain

31 $$\begin{array}{l}-2{a}_{3}{A}_{0}-4{c}_{3}{B}_{0}r+(4{a}_{3}{A}_{2}-({b}_{2}+6{c}_{3}){B}_{1}){r}^{2}\\ +\,\mathop{\sum }\limits_{n=3}^{\infty }\,[(n-1)(n+2){a}_{3}{A}_{n}-((n-1){b}_{2}+2(n+1){c}_{3}){B}_{n-1}]{r}^{n}=0,\\ 2{c}_{2}{B}_{0}+2({c}_{2}{B}_{1}+{b}_{2}{A}_{0})r+(2{c}_{2}{B}_{2}-({a}_{3}-2{a}_{2}+{a}_{1}){B}_{0}+(3{b}_{2}+2{c}_{3}){A}_{1}){r}^{2}\\ +\,\mathop{\sum }\limits_{n=3}^{\infty }\,[2{c}_{2}{B}_{n}-({a}_{3}-2{a}_{2}+{a}_{1}){B}_{n-2}+((n+1){b}_{2}+2(n-1){c}_{3}){A}_{n-1}]{r}^{n}=0.\end{array}\}$$

Equation (31) is satisfied if we choose

32 $${A}_{0}={A}_{2}=0,\,{A}_{1}\ne 0,\,{B}_{0}={B}_{1}=0,\,{B}_{2}=-\,\frac{3{b}_{2}+2{c}_{3}}{2{c}_{2}}{A}_{1},$$

and we can prove the following relations between the constants:

$$\begin{array}{rcl}{A}_{3} & = & -\,\frac{3{b}_{2}+2{c}_{3}}{2{c}_{2}}{W}_{3}{A}_{1},\,{A}_{2n+1}=-\,\frac{3{b}_{2}+2{c}_{3}}{2{c}_{2}}{A}_{1}{W}_{2n+1}\mathop{\prod }\limits_{i=2}^{n}{Q}_{2i}\,n\ge 2,\\ {B}_{2} & = & -\,\frac{3{b}_{2}+2{c}_{3}}{2{c}_{2}}{A}_{1}\,{B}_{2n}=-\,\frac{3{b}_{2}+2{c}_{3}}{2{c}_{2}}{A}_{1}\mathop{\prod }\limits_{i=2}^{n}{Q}_{2i},\,n\ge 2,\end{array}\}$$

with

$$\begin{array}{llll}{Q}_{2i} & = & \frac{{a}_{3}-2{a}_{2}+{a}_{1}}{2{c}_{2}}-\frac{(2i+1){b}_{2}+2(2i-1){c}_{3}}{2{c}_{2}}\frac{(i-1){b}_{2}+4i{c}_{3}}{(i-1)(2i+1){a}_{3}}, & i\ge 2,\\ {W}_{2n+1} & = & \frac{n{b}_{2}+2(n+1){c}_{3}}{n(2n+3){a}_{3}} & n\ge 1.\end{array})$$

Based on the previous mathematical calculations, Eq. (30) takes the form

33 $${{u}}_{{r}}({r})={{A}}_{1}{r}-\frac{3{{b}}_{2}+2{{c}}_{3}}{2{{c}}_{2}}{{W}}_{3}{{A}}_{1}{{r}}^{3}-\frac{3{{b}}_{2}+2{{c}}_{3}}{2{{c}}_{2}}{{A}}_{1}\mathop{\sum }\limits_{{n}=2}^{\infty }({{W}}_{2{n}+1}\mathop{\prod }\limits_{{i}=2}^{{n}}{{Q}}_{2{\boldsymbol{i}}}){{r}}^{2{n}+1},$$

34 $${{s}}_{{rr}}({r})=-\frac{3{{b}}_{2}+2{{c}}_{3}}{2{{c}}_{2}}{{A}}_{1}{{r}}^{2}-\frac{3{{b}}_{2}+2{{c}}_{3}}{2{{c}}_{2}}{{A}}_{1}\mathop{\sum }\limits_{{n}=2}^{\infty }(\mathop{\prod }\limits_{{i}=2}^{{n}}{{Q}}_{2{\boldsymbol{i}}}){{r}}^{2{n}}.$$

A solution to the system of ordinary differential Eq. (27) may be found in series form as in (30). Applying the boundary conditions (28), we find that the first boundary condition is automatically satisfied while the second one gives

$${A}_{1}=\frac{2{c}_{2}}{3{b}_{2}+2{c}_{3}}\frac{{q}_{r}}{2\lambda +{a}_{3}+({a}_{3}-{a}_{2}-(2\lambda +3{a}_{3}){W}_{3}){R}^{2}+{\sum }_{{\boldsymbol{n}}=2}^{\infty }({W}_{2n+1}^{\ast }{R}^{2n}{\prod }_{i=2}^{n}{Q}_{2i})}.$$

with

$${W}_{2n+1}^{\ast }={a}_{3}-{a}_{2}-(2\lambda +(2n+1){a}_{3}){W}_{2n+1}.$$

## Numerical Results

Table 1 shows the values for the physical parameters^23^ for an epoxy matrix and the inclusions materials.

**Table 1:** Phononic material constants.

Radius of the sphere	$R=0.2\,m$
Micro-inertia density	$J=2{d}^{2}/5$
Micro-moduli	${\lambda }_{m}=406\,GPa,\,{\mu }_{m}=273.1\,GPa,$
Macro-moduli	$\lambda =406\,GPa,\,\mu =273.1\,GPa,$
Mass density	${\rho }_{m}=27000\,kg/{m}^{3},\,\rho =2700\,kg/{m}^{3},$
Coupling moduli	${\lambda }_{c}=-\,0.9\,{\lambda }_{e},\,{\mu }_{c}=-\,0.9\,{\mu }_{e},$
Length scales	${\ell }_{1}=0,\,{\ell }_{2}=0.0125\,m,$
the rule of mixture	${\lambda }_{e}=f{\lambda }_{m}+(1-f)\lambda ,$
	$${\mu }_{e}=f{\mu }_{m}+(1-f)\mu ,$$
The applied force	${q}_{r}=30\,N.$

The following figures are obtained depending on the numerical values for materials parameters listed in Tables (1) and (2).

**Table 2:** Radius of the inclusions *d*, number of inclusions and the frequency.

*d*	*M* = *R*/2*d*	*f* = 2 *Md*^3^/*R*^3^
0.01	1000	$2.\times {10}^{-7}$
0.0125	800	$3.91\times {10}^{-7}$
0.016	625	$6\times {10}^{-7}$
0.02	500	$1\times {10}^{-6}$
0.025	400	$1.5625\times {10}^{-6}$
0.04	250	$4\times {10}^{-6}$
0.05,	200	$6.25\times {10}^{-6}$
0.08	125	$0.16\times {10}^{-4}$
0.1	100	$0.25\times {10}^{-4}$
0.2	50	$0.1\times {10}^{-3}$

**Figure 3:**
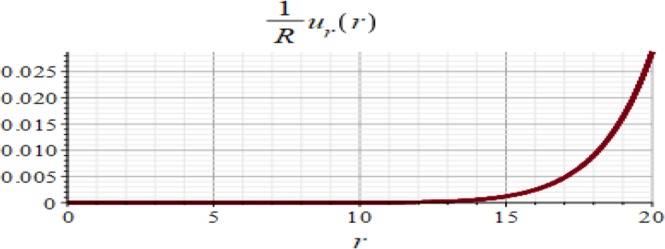
Displacement in *r*-direction when *R* = 20 cm.

**Figure 4:**
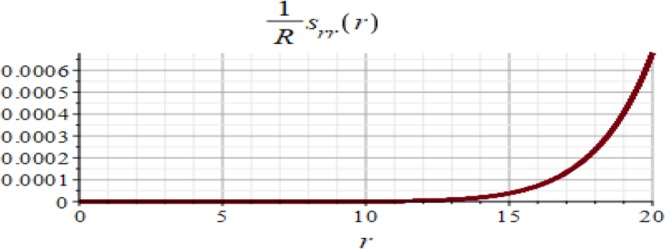
Micro-strain in *r*-direction when *R* = 20 cm.

**Figure 5:**
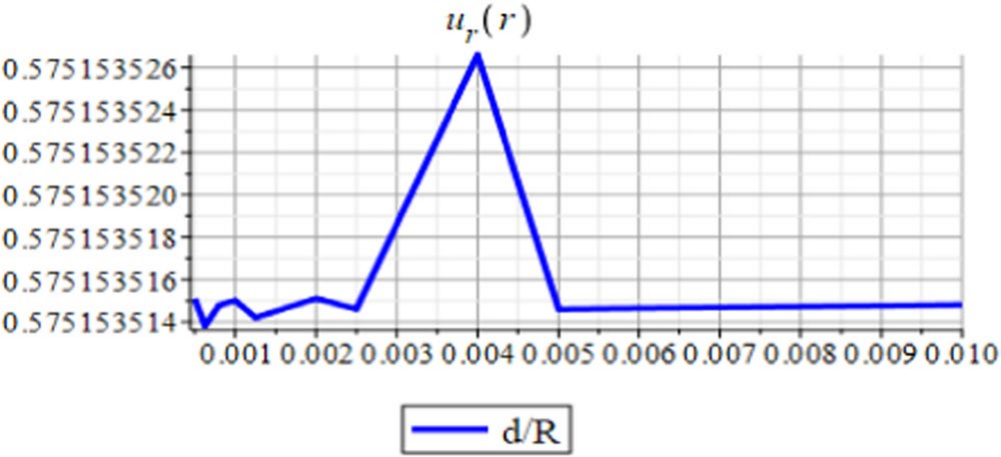
Displacement in *r*-direction with changing $\frac{d}{\mathrm{R.}}$

**Figure 6:**
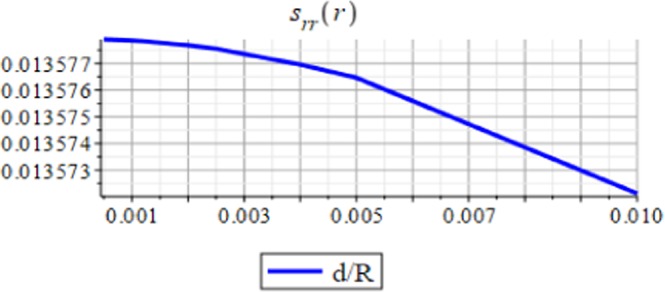
Micro-strain in *r*-direction with changing $\frac{d}{R}$.

Figures (3 and 4) show the displacement and the micro-strain at the hemisphere surface. It is noted that the displacement and the micro-strain increase at the surface. Also, the micro-strain relative to the radius is of order 10^−4^ and the displacement relative to the radius is of order 10^−3^. Figures (5 and 6) show the displacement and the micro-strain for different values for the ratio with changing $\frac{d}{R}$. We note that the displacement increases in a linear way until $\frac{d}{R}=0.004$ then decreases until $\frac{d}{R}=0.005$, then becomes constant. On the other hand, the micro-strain decreases in a nonlinear way with increasing $\frac{d}{R}$.

**Figure 7:**
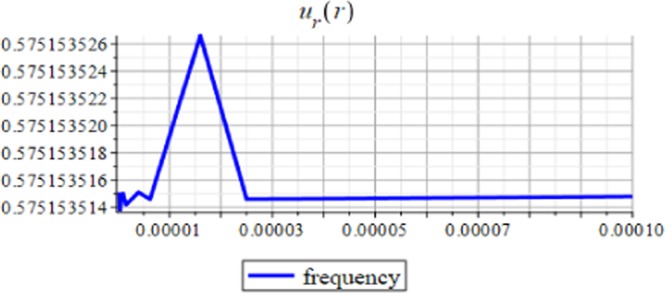
Displacement in *r*-direction for different values of the frequency.

**Figure 8:**
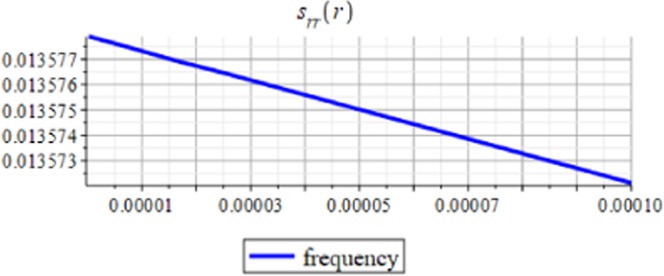
Micro-strain in *r*-direction for different values of the frequency.

Figures (7 and 8) show the displacement and micro-strain with changing with the frequency $f=2M{(\frac{d}{R})}^{3}$ respectively. The change in the displacement can be tangible at the nanoscale (10^−9^), while the change in the micro-strain is linear decreasing and can be tangible at the microscale (10^−6^).

**Figure 9:**
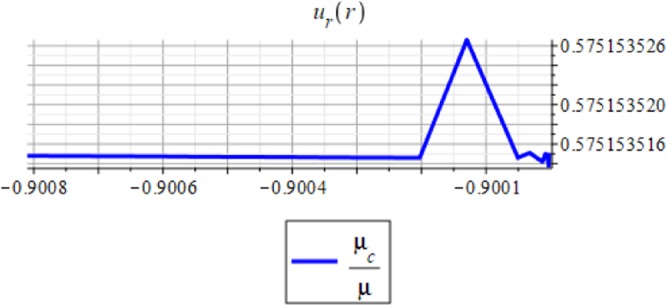
Displacement in *r*-direction for different values of *μ*_*c*_/*μ*.

**Figure 10:**
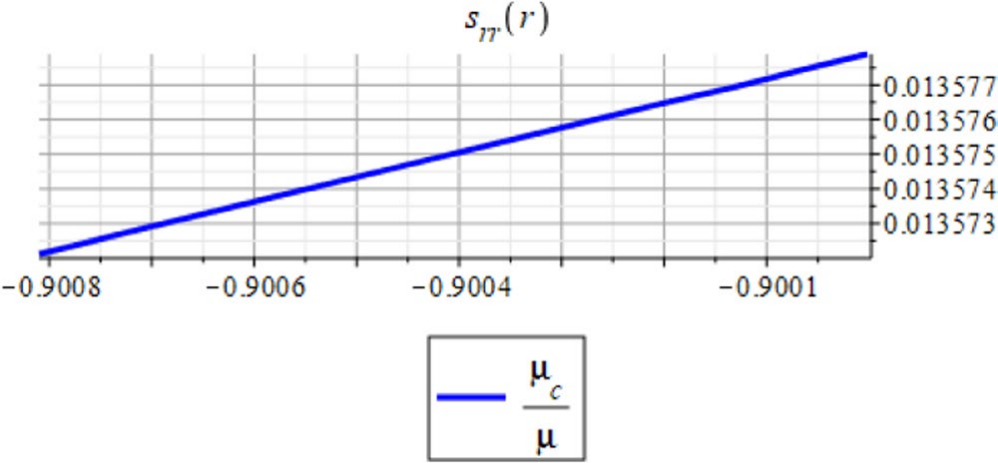
Micro-strain in *r*-direction for different values of *μ*_*c*_/*μ*.

**Figure 11:**
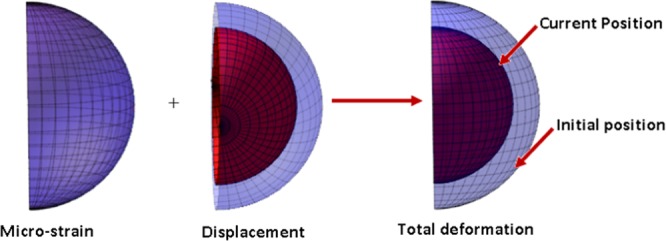
Initial and current positions for the hemisphere.

**Figure 12:**
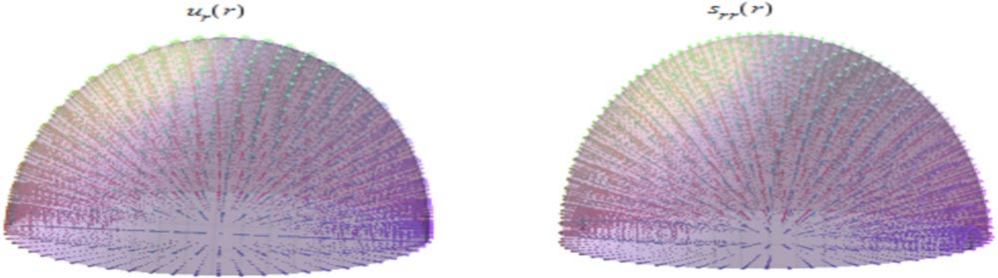
Displacement and micro-strain distribution at surface of the hemisphere.

Figures (9 and 10) show the displacement and micro-strain with changing with the ration *μ*_*c*_/*μ* respectively. The change in the displacement can be tangible at the nanoscale (10^−9^) while the change in the micro-strain is linear increasing and can be tangible at the microscale (10^−6^). Figure (11) show the initial and current position of the hemisphere due to the micro-strain and displacement at the surface, the total deformation of the hemisphere is linear. Note that we measure only the deformation of objects on their surfaces. Figure (12) shows the distribution of the displacement and the micro-strain at the surface of the hemisphere, they are uniformly distributed.

## Concluding Remarks

In this paper, the reduced micromorphic model (RMM) is reformulated and presented in orthogonal curvilinear coordinates. Specific forms for the field equations, boundary conditions, and the constitutive relations have been derived in spherical coordinates. This model may be conveniently applied to a wide range of problems. As an application for this model, a hemisphere made of phononic crystals is considered, where the main unknown functions are the displacement and the micro-strain, changing with the radius of the hemisphere only, and neglecting any dependence on the other coordinates.

The analytical solution is obtained for the field equations using Frobenius series. The unknown coefficients of the considered problem are determined. The results of this study are summarized as follows:

- The displacement and the micro-strain are concentrated at the surface of the hemisphere.
- The displacement increases linearly with the increase of the inclusion radius.
- The micro-strain linearly decreases when $\,d/R\in [0.001,\,0.005]$, and decreases linearly when $d/R\in [0.005,\,0.01].$ In other words, when the number of inclusions is 100 or 50, the change in micro-strain is linearly decreasing.
- The displacement has a greater effect than the micro-strain, when it is measured relative to the classical physical quantities as on Figs. (3 and 4).
- The micro-strain has a greater effect than the displacement when it is measured relative to the nanoscale physical quantities as on Figs. (5–10).
